# Epidemiology of hepatitis A in Saudi Arabia: a retrospective analysis of Ministry of Health surveillance and yearbook data, 2006–2023

**DOI:** 10.3389/fepid.2026.1717102

**Published:** 2026-03-30

**Authors:** Ibrahim G. Alghamdi

**Affiliations:** Public Health Department, College of Applied Medical Sciences, University of AL-Baha, AL-Baha, Saudi Arabia

**Keywords:** epidemiology, hepatitis A virus, incidence, retrospective study, Saudi Arabia, vaccination, joinpoint regression, surveillance

## Abstract

**Background:**

This study investigates the epidemiology of hepatitis A virus (HAV) infection in Saudi Arabia from 2006 to 2023, focusing on temporal, demographic, and geographic variations in incidence. The aim was to characterize long-term national trends and identify high-risk subgroups to inform prevention strategies.

**Methods:**

National HAV surveillance data were obtained from the Saudi Ministry of Health Statistical Yearbooks. Crude incidence rates (CIRs) were calculated using mid-year population estimates. Temporal trends were assessed via Joinpoint regression to estimate annual percent change (APC) and average annual percent change (AAPC). Regional differences were analyzed with negative binomial regression using Riyadh as the reference. Group comparisons employed nonparametric tests, with statistical significance set at *α*=0.05.

**Results:**

Between 2006 and 2023, 9,820 HAV cases were reported (mean 546/year). National CIR declined from 11.1 per 100,000 in 2006 to 0.48 in 2023, with an AAPC of −19.5% (95% CI −24.2 to −14.4; *p* < 0.001). Children aged 5–14 years bore the highest burden (53.5% of cases). Significant regional heterogeneity was observed, with persistently higher CIRs in Najran and Qurayyat, while urban centers showed lower, stable rates. Negative binomial regression identified higher adjusted risks in Qurayyat (IRR 2.89) and Najran (IRR 2.52). Saudis initially showed higher incidence than non-Saudis, but rates converged by 2023. Males consistently outnumbered females (ratio ∼1.6).

**Conclusion:**

HAV incidence in Saudi Arabia has markedly declined over the past two decades, reflecting improved sanitation and public health measures. This decline may, in part, reflect the impact of the national hepatitis A vaccination program introduced in 2008. However, age-, region-, and nationality-specific disparities remain, underscoring the need for geographically tailored interventions and consideration of targeted vaccination strategies to sustain progress and prevent resurgence.

## Introduction

Hepatitis A is an acute viral liver disease caused by the hepatitis A virus (HAV), an enterically transmitted pathogen primarily spread through ingestion of contaminated food or water and person-to-person contact in settings of poor hygiene (1). Unlike hepatitis B and C, HAV infection does not lead to chronic liver disease; however, acute cases can result in severe illness, hospitalization, and, in rare instances, fulminant hepatic failure, particularly among adults and immunocompromised individuals (2). Effective inactivated vaccines are available and have proven highly protective, but HAV remains a major global public health concern due to uneven vaccine uptake, socioeconomic disparities, and the persistent risk of outbreaks (3).

Globally, the World Health Organization estimates that approximately 159 million HAV infections occur annually, with more than 7,000 related deaths, predominantly in low- and middle-income countries (4). Recent evidence indicates a shift in epidemiological patterns: as sanitation and living standards improve, HAV endemicity decreases, leading to a growing proportion of susceptible adolescents and adults who are at higher risk of symptomatic disease and outbreaks (5). Regional heterogeneity remains significant, with high prevalence in parts of South Asia, Africa, and Latin America, while countries in the Gulf region, including Saudi Arabia, have transitioned toward intermediate-to-low endemicity (6).

In Saudi Arabia, HAV incidence has markedly declined over the past two decades, reflecting national improvements in sanitation, food safety, and healthcare systems (7). Nonetheless, surveillance reports highlight persistent age- and region-specific disparities, with school-aged children and certain peripheral regions bearing disproportionate burdens (8). Furthermore, differences between Saudi nationals and non-nationals underscore the role of socioeconomic and occupational factors in HAV transmission (9).

This study aims to provide a comprehensive assessment of HAV epidemiology in Saudi Arabia from 2006 to 2023. We analyze national surveillance data to describe temporal trends, age- and sex-specific distributions, regional heterogeneity, and differences by nationality, with the goal of informing evidence-based policies to sustain progress and guide prevention strategies.

## Methods

### Study design and population

This retrospective descriptive study analyzed all officially reported hepatitis A virus (HAV) cases in Saudi Arabia from January 2006 through December 2023. Data were aggregated at the calendar-year level across the 20 administrative regions. Analyses were stratified by age (for 2023 only), sex, and nationality where data were available.

### Data sources and case definition

Annual HAV case counts and population denominators were obtained from the publicly available *Statistical Yearbooks* issued by the Saudi Ministry of Health. Population denominators by year and region were available for the full study period (2006–2023). Age-specific denominators were reported only for 2023, which allowed the calculation of age-stratified incidence rates for that year. Data on sex and nationality were directly available in the Yearbook and therefore used as reported. The Ministry of Health compiles these surveillance data annually from public and private healthcare facilities across all administrative regions. However, the database does not include individual-level information such as patients' place of residence (urban or rural), which limits assessment of geographic and sociodemographic risk factors. HAV case definitions followed the Ministry's operational surveillance criteria. Because only de-identified, aggregate data were analyzed, ethical approval and informed consent were not required.

### Rate calculations

For each year and region we obtained the corresponding population denominators from the Statistical Yearbook. We calculated crude incidence rates per 100,000 as the annual case count divided by the mid year population multiplied by 100,000. Age specific incidence rates were calculated for 2023 only using the Yearbook strata 0 to 4, 5 to 14, 15 to 44, and 45 years and over. For each stratum, the rate was computed as cases divided by the corresponding population multiplied by 100,000 and reported to one decimal place (10). An all ages rate was calculated from the total number of cases and the total population in 2023 rather than averaging stratum specific rates.

### Formulas

- Crude incidence rate per 100,000 for year *y* and region *r*:
  CIR{y,r}=(cases{y,r}/population{y,r})×100,000
- Age specific incidence rate for 2023 only for age stratum:
  AgespecificIR{2023}=(cases{2023}/population{2023})×100,000
- All ages in 2023:
  CIR{all,2023}=(Σcases{2023}/Σpopulation{2023})×100,000

### Statistical analysis

Descriptive statistics summarized annual case counts and CIRs overall and across available demographic and regional strata. The Shapiro–Wilk test assessed normality. Group comparisons used t-tests or ANOVA for normally distributed variables and Mann–Whitney U or Kruskal–Wallis tests otherwise. Statistical significance was defined at *α* = 0.05 (two-sided).

Joinpoint regression (NCI Joinpoint Regression Program) was used to model log-transformed CIRs against year, estimating annual percent change (APC) and average annual percent change (AAPC) with 95% confidence intervals. Optimal joinpoints were determined by permutation testing.

Regional heterogeneity was further assessed using negative binomial regression with log-link and population offset, setting Riyadh as the reference. Incidence rate ratios (IRRs) with 95% confidence intervals were reported. Model adequacy was evaluated via deviance and Pearson residuals.

### Software

All analyses were performed using SPSS version 20 (IBM Corp., Armonk, NY, USA). Figures and tables were generated directly from analytic outputs to ensure reproducibility.

## Results

### Annual trends in HAV cases and incidence rate

**Figure 1:**
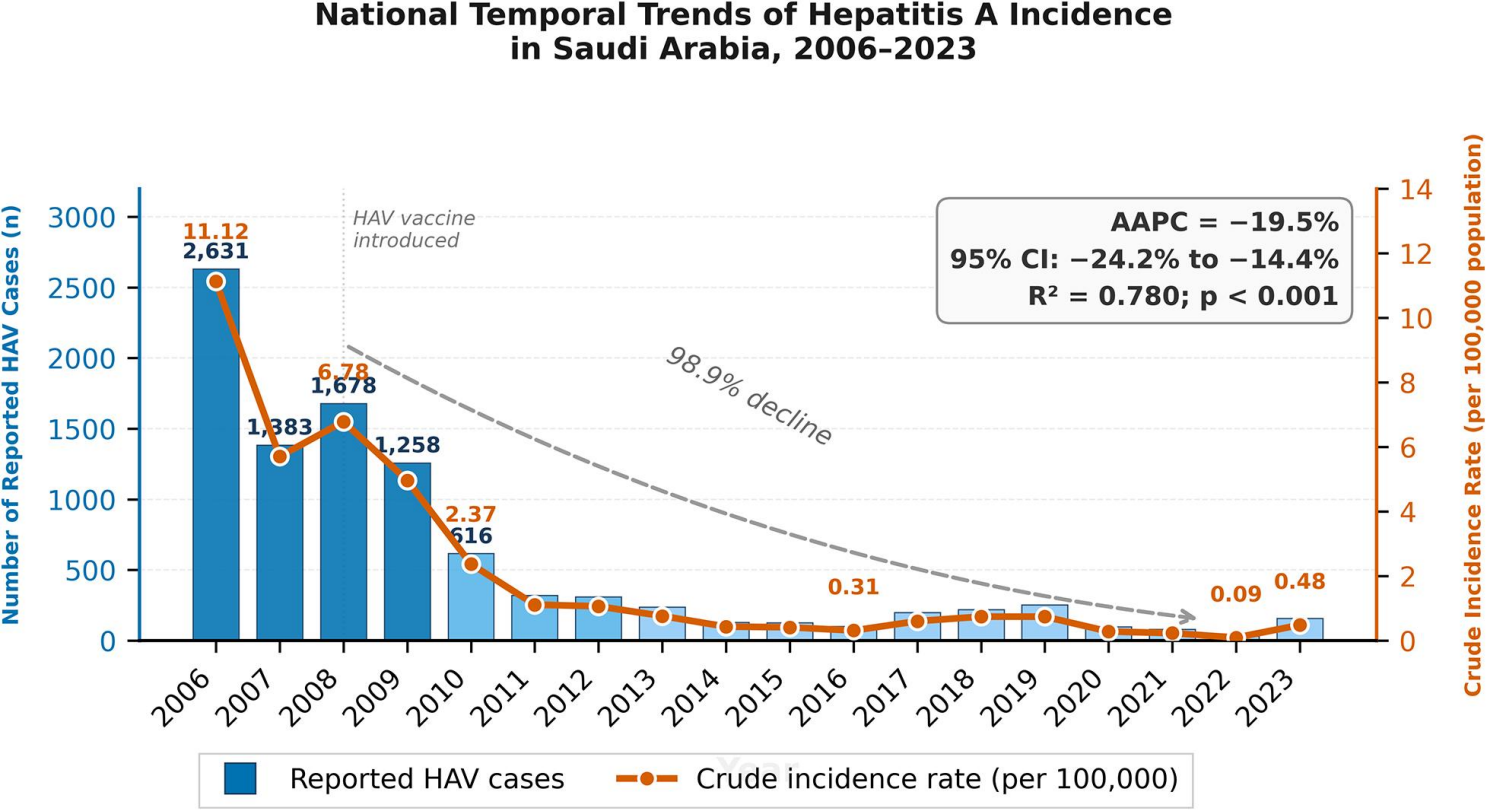
National temporal trends of hepatitis A incidence (2006–2023) in Saudi Arabia.

From 2006 to 2023, a total of 9,820 hepatitis A cases were reported in Saudi Arabia, with a mean of 546 and a median of 228 cases per year. The annual number of cases declined steadily from 2,631 in 2006 [crude incidence rate (CIR) = 11.12 per 100,000] to 321 in 2011 (CIR = 1.11 per 100,000). Between 2012 and 2016, case numbers remained consistently low, ranging from 100 to 310 cases annually, corresponding to CIRs between 0.31 and 1.06 per 100,000. A modest increase was observed between 2017 and 2019 (198–252 cases; CIRs: 0.60–0.74 per 100,000), followed by the lowest counts during 2020–2022 (97, 79, and 30 cases; CIRs: 0.28, 0.23, and 0.09 per 100,000, respectively). In 2023, there were 157 reported cases, corresponding to a CIR of 0.48 per 100,000. Over the entire study period, the mean CIR was 2.10 per 100,000 (median: 0.70 per 100,000). A joinpoint regression analysis revealed a significant average annual percentage change (AAPC) of −19.5% (95% CI: −24.2 to −14.4; *p* < 0.001; R^2^ = 0.780), indicating a sustained decline in crude incidence over time (Figure 1).

### Age distribution of HAV cases and incidence rate

The burden of disease was unequally distributed across age groups, with the highest proportion observed among children aged 5–14 years, accounting for over half of all reported cases (*n* = 5,257; 53.5%; mean = 292 cases/year). This was followed by individuals aged 15–44 years, who represented 26.6% of cases (*n* = 2,615; mean = 145/year), and children aged 1–4 years with 15.0% (*n* = 1,477; mean = 82/year). In contrast, infants under one year and adults aged ≥45 years contributed only 1.1% and 3.7% of the total burden, respectively. Median case counts and annual variability (IQR) highlighted marked fluctuations over time, particularly within the 5–14 age group (IQR = 290) (Supplementary Table S1).

In 2023, the crude incidence rate (CIR) of HAV was 0.4 per 100,000 population, based on 157 reported cases out of a total population of 35.3 million. The highest age-specific incidence was recorded among adults aged 15–44 years (0.5 per 100,000; 115 cases) and children aged 0–4 years (0.5 per 100,000; 12 cases), followed by children aged 5–14 years (0.4 per 100,000; 21 cases). The lowest incidence was noted among those aged ≥45 years (0.1 per 100,000; 9 cases). Additionally, a Kruskal–Wallis test demonstrated significant differences in annual HAV case distributions across the five age groups [*χ*^2^(4, *N* = 90) = 46.32, *p* < 0.001], with *post-hoc* analysis revealing that the <1-year group differed significantly from all others, while no difference was observed between the 1–4 and 5–14-year groups.‏ These findings demonstrate the distribution of reported HAV cases by age group and the relative burden across the population (Supplementary Table S1).

### Regional HAV cases & incidence rate

The Riyadh region accounted for the highest cumulative burden, representing 14.4% of total cases (*n* = 1,419), with a yearly mean of 78.8 and a median of 61.5 cases (IQR = 62.2). Makkah (9.2%; *n* = 907) and Jeddah (8.9%; *n* = 875) followed, both exhibiting considerable inter-annual variability, particularly Makkah (IQR = 67.2). Comparable case volumes were reported from Taif (8.7%; *n* = 855) and Madinah (8.2%; *n* = 809), with Madinah showing the highest interquartile range (IQR = 83.8), reflecting significant year-to-year fluctuations. Moderate cumulative burdens were reported from Qassim (7.8%; *n* = 763), the Eastern Region (7.0%; *n* = 689), and Al-Ahsa (7.0%; *n* = 686), each characterized by relatively consistent annual figures. In contrast, regions including Bishah, Tabuk, Hail, and the Northern Region contributed fewer cases (2.3%–3.0% of total), with low medians and narrow IQRs, indicating limited or sporadic transmission. The lowest case counts were recorded in AL-Baha, Jouf, Qurayyat, and Qunfudah, jointly comprising less than 3% of total cases, and demonstrating median values near zero and minimal interquartile variation (Supplementary Table S2).

**Figure 2:**
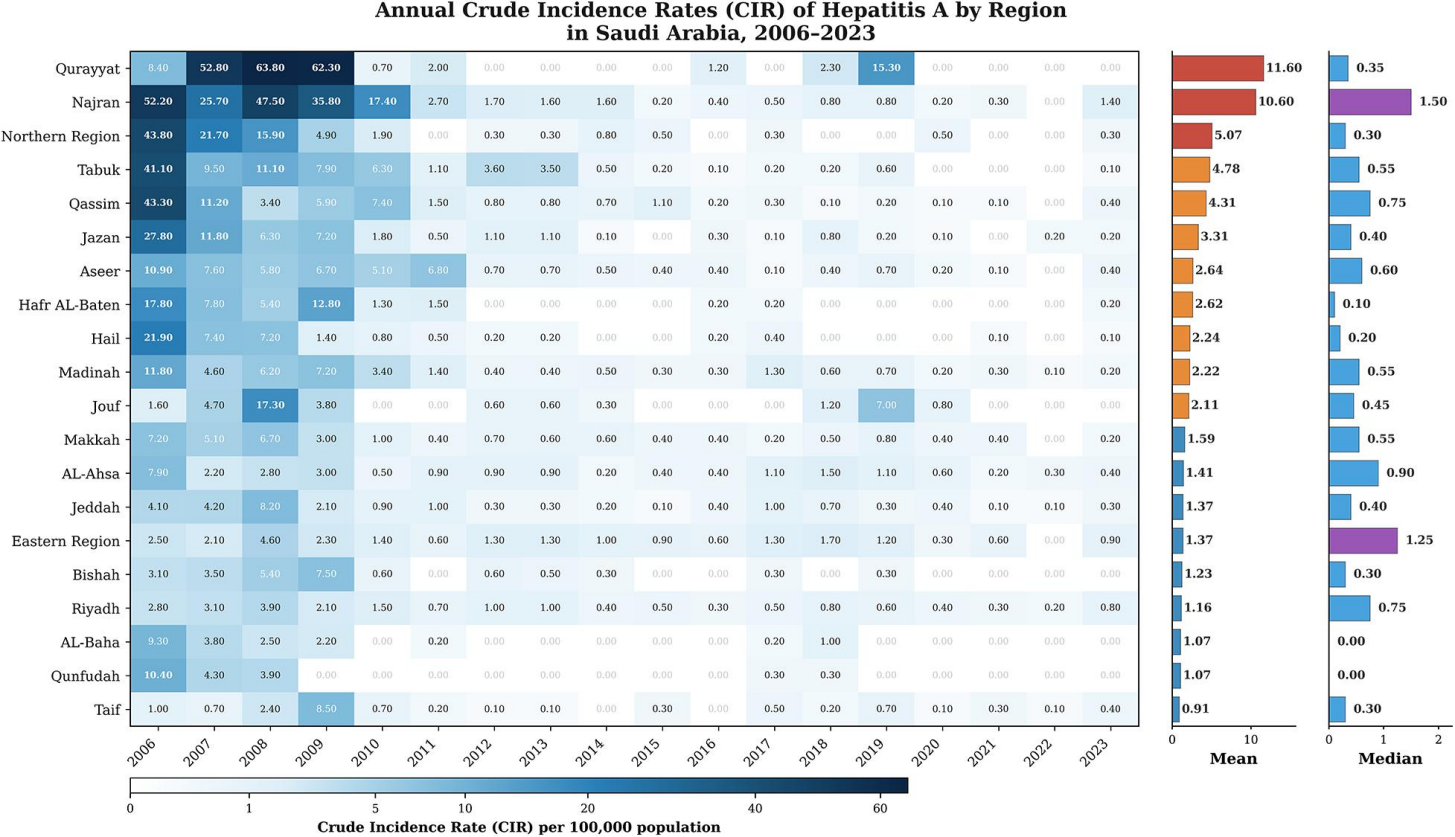
Annual crude incidence rates (CIRs) of hepatitis A by region in Saudi Arabia (2006–2023).

Crude incidence rates (CIRs) of hepatitis A demonstrated wide regional disparities over the 18-year period, as visualized in the heatmap (Figure 2). Regions with consistently elevated CIRs included Najran, Qurayyat, Northern Region, Qassim, and Tabuk, each recording multiple years with CIRs exceeding 10 per 100,000. Notably, Najran peaked at 52.2 in 2006, Qurayyat exceeded 60 in multiple years (2008–2009), and Qassim reported a maximum CIR of 43.3. By contrast, central and urban regions such as Riyadh, Makkah, and Jeddah showed lower and more stable CIRs, particularly in recent years. Several peripheral regions including Qunfudah, Jouf, and AL-Baha maintained low CIRs throughout the study period. Mean CIRs over the 18-year period were highest in Najran (10.6), Qurayyat (11.6), and Qassim (4.3), compared to national urban centers such as Riyadh (1.2) and Jeddah (1.4). Median CIRs were ≤1.5 per 100,000 in the majority of regions, reflecting the declining trend in national HAV incidence. A Kruskal–Wallis analysis confirmed significant differences in CIR distributions across regions [*χ*^2^(19, *N* = 360) = 46.72, *p* = 0.0004], reinforcing the geographic heterogeneity of HAV transmission in Saudi Arabia.

### Joinpoint analysis of regional HAV CIR (2006–2023)

**Figure 3:**
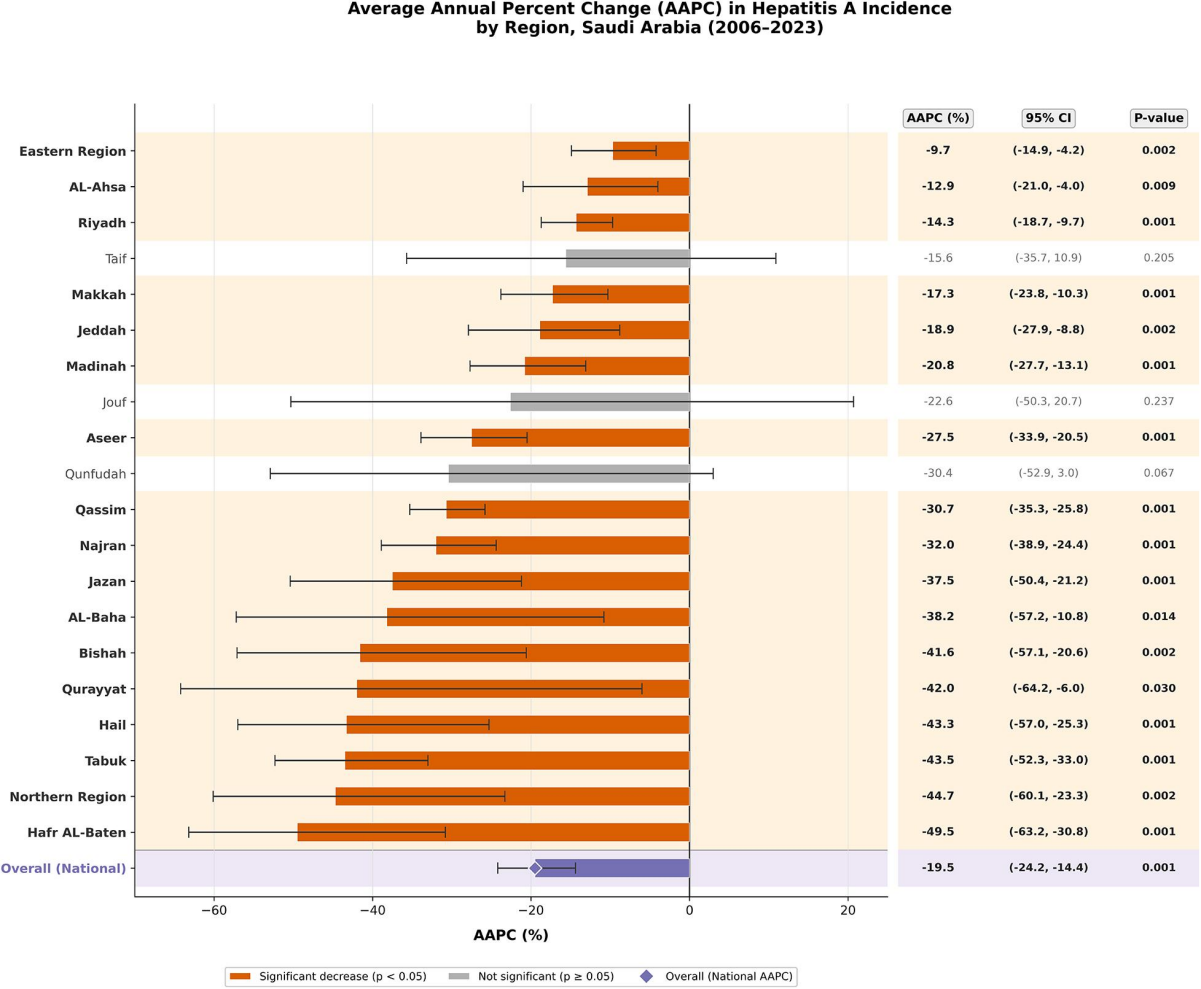
Average annual percent change (AAPC) in hepatitis A incidence by region (2006–2023).

Joinpoint regression analysis revealed statistically significant declines in hepatitis A crude incidence rates (CIRs) across the majority of Saudi regions between 2006 and 2023, with marked regional variability in the average annual percentage change (AAPC). Among regions with significant downward trends, the sharpest declines were observed in Hafr AL-Baten (AAPC = −49.5%; 95% CI: −63.2 to −30.8), Northern Region (−44.7%), Tabuk (−43.5%), Hail (−43.3%), and Qurayyat (−42.0%), all with *p* < 0.05 (Figure 3). Other regions with notable significant reductions include Najran (−32.0%), Qassim (−30.7%), Aseer (−27.5%), Madinah (−20.8%), and Jeddah (−18.9%). Urban centers such as Riyadh, Makkah, and Eastern Region also experienced significant but more modest declines (−9.7% to −17.3%). In contrast, Taif, Jouf, and Qunfudah showed no statistically significant trends over the study period, despite point estimates indicating declining AAPCs.

### Negative binomial regression of regional HAV case counts (Riyadh reference)

**Figure 4:**
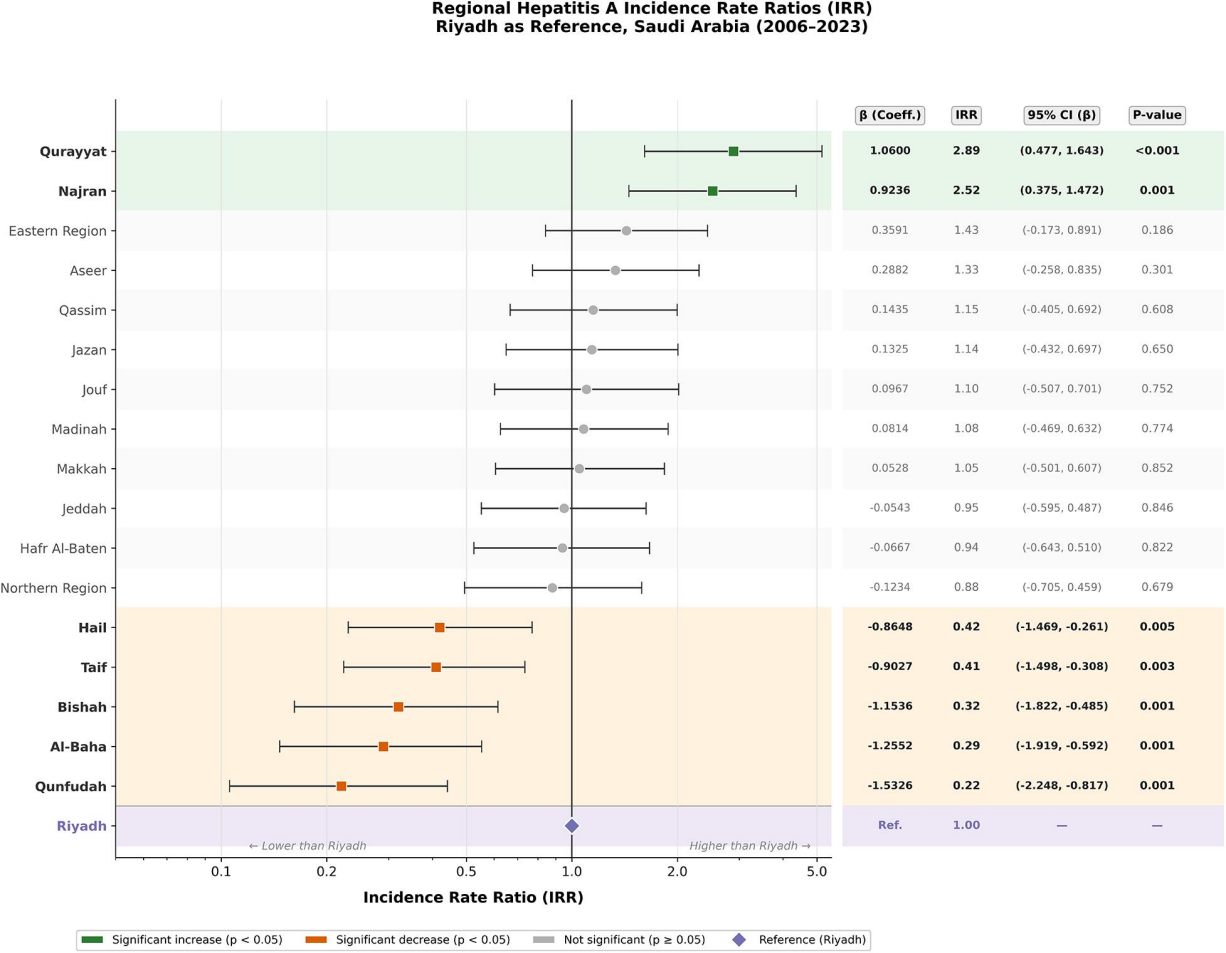
Regional incidence rate ratios (IRRs) of hepatitis A compared with Riyadh (reference), 2006–2023.

A negative binomial regression model was applied to evaluate regional differences in hepatitis A virus (HAV) case counts using Riyadh as the reference group. The model identified two regions with significantly higher incidence rate ratios (IRRs) relative to Riyadh: Qurayyat (IRR = 2.89; 95% CI: 1.61–5.17; *p* = 0.0004) and Najran (IRR = 2.52; 95% CI: 1.46–4.35; *p* = 0.001), indicating nearly threefold increases in reported case counts. In contrast, five regions exhibited significantly lower incidence: Qunfudah (IRR = 0.22; 95% CI: 0.11–0.44; *p* < 0.001), Al-Baha (IRR = 0.29; 95% CI: 0.15–0.55), Bishah (IRR = 0.32; 95% CI: 0.16–0.61), Taif (IRR = 0.41; 95% CI: 0.22–0.73), and Hail (IRR = 0.42; 95% CI: 0.23–0.77), all with *p* < 0.01. The remaining regions including Eastern Region, Jeddah, Makkah, Madinah, and Qassim showed IRRs not significantly different from Riyadh, indicating comparable adjusted HAV burden across these populations (Figure 4).

### Nationality HAV cases & incidence rate

**Figure 5:**
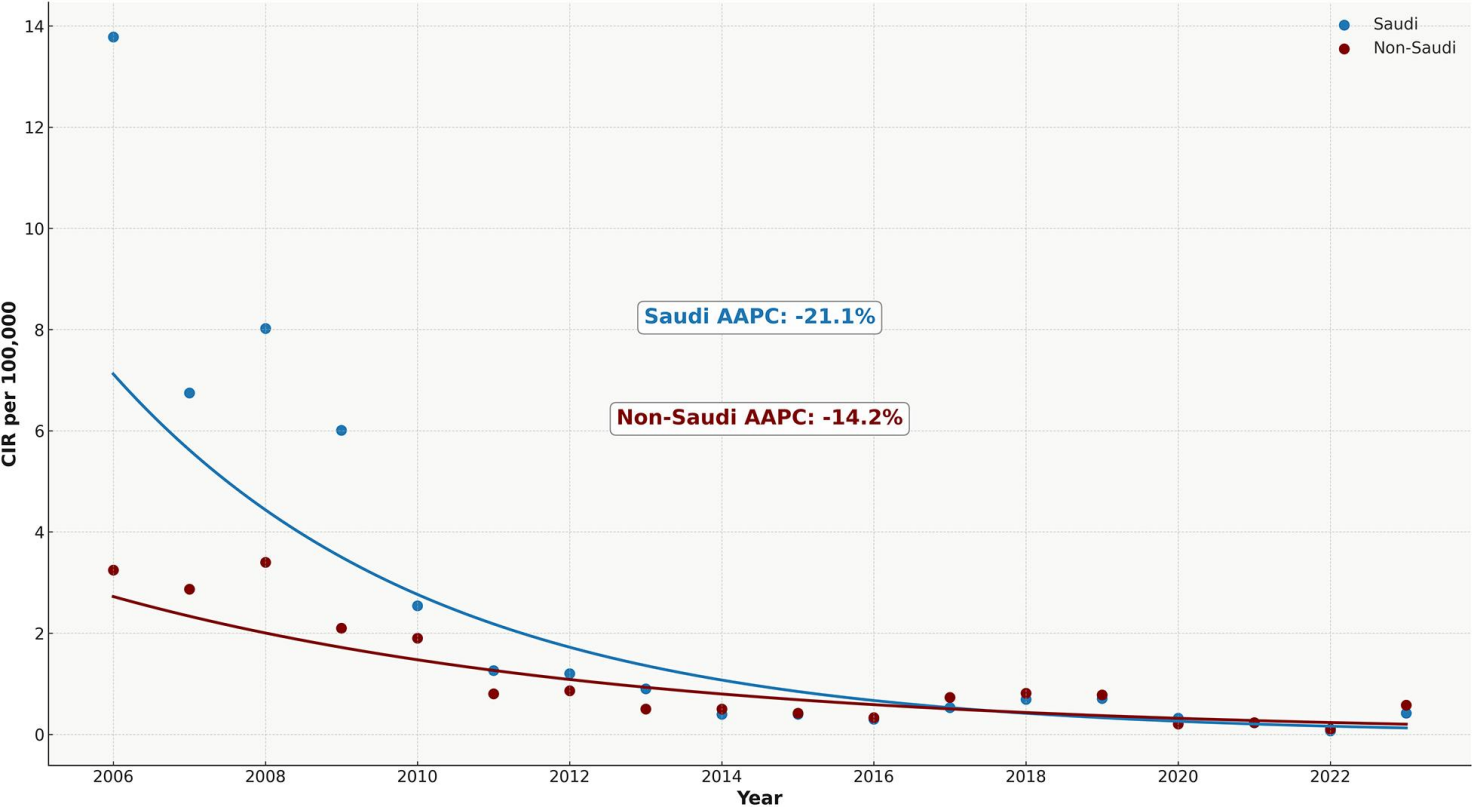
Comparative temporal trends of hepatitis A incidence among Saudi and non-Saudi populations (2006–2023).

Between 2006 and 2023, a total of 8,193 hepatitis A cases were reported among Saudi nationals and 1,627 among non-Saudis. Among Saudis, the annual crude incidence rate (CIR) showed a marked decline, with a mean CIR of 2.5 per 100,000 and a median of 0.7 per 100,000, reflecting the skewed distribution driven by higher incidence in the early years. Among non-Saudis, the CIR was consistently lower, with an overall mean of 1.1 and a median of 0.8 per 100,000. Males accounted for a higher number of cases across both groups, with an overall male-to-female ratio of 1.7 in Saudis and 1.5 in non-Saudis. While Saudi nationals exhibited higher CIRs in the earlier part of the study period, this difference steadily declined over time (Supplementary Table S3). Joinpoint regression analysis confirmed statistically significant downward trends in both populations, with a sharper average annual percentage change (AAPC) among Saudis (−21.1%) compared to non-Saudis (−14.2%) (Figure 5). The CIR ratio between Saudis and non-Saudis decreased from 4.2 in 2006 to 0.7 in 2023, signalling a convergence in disease burden. Notably, in the latter years of the surveillance period, non-Saudis at times recorded comparable or higher CIRs than Saudis.

## Discussion

Hepatitis A continues to pose a significant public health challenge in Saudi Arabia, particularly given its potential for outbreaks and its impact on both citizens and non-citizen populations. Addressing this challenge requires ongoing surveillance and data-driven strategies to guide prevention and control efforts. The present study represents a critical contribution to national hepatitis A knowledge, offering one of the most extensive epidemiological analyses of HAV incidence trends over nearly two decades. By highlighting the marked decline in incidence, demographic disparities, and evolving patterns across nationality and region, these findings provide valuable insights to support more equitable and targeted public health interventions, ultimately enhancing the resilience of national hepatitis A prevention programs.

### Annual trend pattern of HAV in Saudi Arabia

The significant and sustained decline in hepatitis A virus (HAV) incidence in Saudi Arabia from 2006 to 2023 reflects the impact of progressive public health interventions and improved sanitation infrastructure. The average annual percentage change (AAPC) of −19.5% (*p* < 0.001) indicates a clear downward trajectory in HAV burden, consistent with global trends in countries undergoing socioeconomic transition and improved access to clean water and hygiene facilities (11). The sharp decline during the early years (2006–2011), followed by relatively low endemicity from 2012 onwards, aligns with the period of intensified national vaccination programs, particularly the introduction of HAV immunization in high-risk areas and among children (12). The national introduction of hepatitis A vaccination in 2008 likely played a substantial role in the observed decline in HAV incidence. The inclusion of HAV immunization within the Saudi Expanded Program on Immunization, initially targeted at children and high-risk groups, has contributed to sustained reductions in community transmission. This aligns with international evidence that early childhood vaccination effectively reduces both symptomatic cases and outbreaks in transitional settings. The observed increase between 2017 and 2019 may reflect localized outbreaks or gaps in herd immunity, while the sharp reduction during 2020–2022 may have been influenced by COVID-19 pandemic-related restrictions, which limited viral transmission through improved hygiene and reduced mobility (13). Nevertheless, the modest rebound in 2023 underscores the need for ongoing surveillance and vaccination efforts. These findings support the transition of HAV epidemiology in Saudi Arabia from intermediate to low endemicity, necessitating updated immunization policies and outbreak preparedness (14).

### Age-stratified HAV patterns

The age-specific distribution of HAV in Saudi Arabia between 2006 and 2023 reveals a disproportionately high burden among children aged 5–14 years, who accounted for more than half of all cases. This pattern is consistent with HAV epidemiology in transitional countries, where shifting from high to intermediate or low endemicity increases the average age of infection, resulting in greater clinical recognition among school-aged children (11). The observed high incidence in this group suggests continued fecal-oral transmission within school and household settings, highlighting a potential gap in targeted vaccination or hygiene education (6). Although universal hepatitis A vaccination is not yet implemented in Saudi Arabia, the data suggest that targeted immunization strategies for children especially before school entry may offer significant public health benefits (12). In contrast, infants and adults over 45 years consistently demonstrated low incidence, likely due to a combination of maternal antibody protection in infants and past natural exposure or lower risk behaviors among older adults (14). The significant intergroup differences revealed by the Kruskal–Wallis test underscore the need for age-specific public health responses. Surveillance and education strategies tailored to school-age children could enhance prevention efforts and mitigate future outbreaks.

### Spatial-temporal heterogeneity in HAV patterns

The pronounced regional disparities in hepatitis A incidence across Saudi Arabia reflect complex interactions between geography, population density, sanitation standards, and local public health infrastructure. Peripheral regions such as Qurayyat, Najran, and Qassim consistently recorded higher crude incidence rates (CIRs), exceeding 10 per 100,000 in several years, while central urban centers like Riyadh and Jeddah showed markedly lower and more stable trends. These findings align with international observations that HAV transmission is often sustained in less urbanized or underserved regions, where sanitation and access to clean water may lag behind (5). The significant spatial heterogeneity confirmed by statistical analyses (*p* < 0.001) underscores the importance of region-specific interventions. Areas with persistently high incidence could benefit from localized vaccination campaigns and improvements in hygiene infrastructure (10). Temporal analysis further revealed variable joinpoint trends across regions, with some experiencing steep annual declines (e.g., Hafr Al-Baten, Qurayyat), while others like Qunfudah and Taif showed no significant changes highlighting potential gaps in intervention coverage (14). These spatial-temporal patterns emphasize the need for dynamic and geographically tailored HAV control strategies rather than a uniform national approach (15). These findings are also supported by previous national research. Al Faleh et al. (2008) reported a marked decline in anti-HAV antibody prevalence among Saudi children and adolescents over an 18-year period, attributing this decrease to improvements in living standards and hygiene practices in Saudi Arabia (16). Similarly, Sharaheeli and Alibrahim (2022) analyzed 11,148 confirmed foodborne hepatitis A cases reported to the Saudi Ministry of Health between 2005 and 2015. They concluded that foodborne transmission remains a relevant route of HAV infection in Saudi Arabia and that regional variations likely reflect differences in food safety practices, hygiene, and surveillance systems (17). Together, these earlier studies complement the present findings by illustrating both the long-term epidemiological transition and the residual outbreak potential in specific settings.

### Nationality and sex pattern of HAV incidence rate

The observed differences in HAV incidence between Saudi nationals and non-Saudis over the 18-year period reveal dynamic epidemiological trends shaped by demographics, living conditions, and healthcare access. Initially, Saudis exhibited markedly higher crude incidence rates (CIRs), but a convergence occurred over time, with the CIR ratio dropping from 4.2 in 2006 to 0.7 in 2023. This shift may reflect improved HAV awareness, vaccination outreach, and sanitation among the native population, coupled with increased risk among non-Saudis, particularly labor migrants, who may reside in crowded housing with limited access to preventive healthcare (18). The consistently higher male-to-female ratio in both groups (1.7 in Saudis, 1.5 in non-Saudis) aligns with established patterns where men especially working-age adults are more frequently exposed to occupational or environmental risk factors for HAV transmission (19). In addition, greater male mobility and more frequent social mixing in community and work environments may increase opportunities for HAV exposure in Saudi Arabia, further contributing to the higher incidence observed among males. These findings underscore the importance of tailoring HAV prevention programs to migrant and male-dominated populations, especially in occupational settings where sanitation may be suboptimal (15). Furthermore, surveillance systems should be strengthened to capture real-time shifts in risk distribution between population groups, enabling proactive, equitable public health interventions.

### Global patterns of hepatitis A prevalence rates

Globally, hepatitis A virus (HAV) prevalence exhibits stark contrasts aligned with countries' socioeconomic status, sanitation infrastructure, and public health policies. High endemicity remains prevalent in many parts of sub-Saharan Africa, South Asia, and Latin America, where poor access to clean water and sanitation facilitates early childhood exposure, often resulting in asymptomatic infection and natural immunity acquisition (6). In contrast, low endemicity is observed in high-income countries across North America, Western Europe, and the Gulf Cooperation Council (GCC) states, including Saudi Arabia in recent years, where improved living conditions and hygiene practices have shifted the epidemiological profile (11). However, this shift toward low endemicity paradoxically increases population susceptibility to symptomatic adult infections and outbreaks, particularly in the absence of universal immunization (12). Recent global surveillance has revealed emerging HAV outbreaks in low-endemic settings among non-immune adults, migrants, and specific risk groups such as men who have sex with men (MSM), indicating that complacency in vaccination policies can reverse progress (20). Saudi Arabia's declining incidence pattern mirrors this global trend but also highlights a transitional risk landscape marked by urban-rural disparities, shifting age distributions, and growing vulnerability among unvaccinated adults and mobile populations. Consequently, national HAV control strategies must integrate local epidemiological trends within a broader global context to mitigate future resurgence and ensure sustained progress.

This study has several limitations that warrant consideration when interpreting its findings. It is important to note that in Saudi Arabia, hepatitis A and other notifiable infectious diseases are mandatorily reported to the Ministry of Health from both public and private healthcare sectors, including hospitals, laboratories, and clinics. Consequently, data from private facilities are incorporated into the national surveillance system and summarized annually in the Statistical Yearbook. However, variations in reporting completeness between sectors and regions may still exist and could contribute to underestimation of true incidence rates. First, as a retrospective descriptive analysis relying on national surveillance data, it is inherently subject to underreporting and reporting bias. Asymptomatic or mild cases particularly in younger age groups may go undetected and unrecorded, potentially underestimating the true incidence of hepatitis A. Second, the quality and completeness of regional and demographic data may vary across provinces and over time, which could introduce inconsistencies in spatial or temporal comparisons. Third, the study lacked access to individual-level data, including vaccination history, socioeconomic status, or exposure risk factors, limiting the ability to identify specific determinants of infection or assess vaccine effectiveness. Fourth, variations in diagnostic criteria, testing availability, and public health reporting practices between regions may have influenced observed incidence rates. Finally, the use of crude incidence rates, rather than age-adjusted or population-weighted measures, may introduce demographic distortions in trend interpretation. Despite these limitations, the study provides valuable insights into the long-term epidemiological transition of hepatitis A in Saudi Arabia and highlights areas for improved data collection and targeted public health strategies in the future.

## Conclusion

This nationwide analysis of hepatitis A trends in Saudi Arabia from 2006 to 2023 reveals a marked decline in incidence, indicating a transition from intermediate to low endemicity. However, age-specific, regional, and demographic disparities persist, particularly among school-aged children and certain peripheral regions. These findings underscore the importance of enhancing surveillance, considering targeted vaccination strategies, and addressing regional inequities in sanitation and healthcare access. As Saudi Arabia continues its public health transformation, sustained efforts are needed to prevent HAV resurgence and to guide evidence-based immunization policies that reflect evolving epidemiological patterns.

## Data Availability

Publicly available datasets were analyzed in this study. This data can be found here: https://www.moh.gov.sa/en/Ministry/Statistics/book/Pages/default.aspx.

## Supplementary material

Supplementary Table S1 Age-specific distribution of reported hepatitis A virus (HAV) cases in Saudi Arabia, 2006–2023, with mean, median, interquartile range (IQR), and percentage of total cases across age groups. Crude incidence rates (CIR) per 100,000 population are shown separately for 2023.

Supplementary Table S2 Regional distribution of reported hepatitis A virus (HAV) cases across Saudi Arabia's administrative regions, 2006–2023. Data include annual counts, totals, mean, median, and interquartile range (IQR) for each region.

Supplementary Table S3 Annual distribution of hepatitis A virus (HAV) cases and crude incidence rates (CIR) in Saudi Arabia, 2006–2023, stratified by nationality (Saudi vs. non-Saudi) and gender. Data include male-to-female ratios, Saudi-to-non-Saudi ratios, and summary measures (totals, overall averages, and medians).
